# Investigation of Barriers and Facilitators to Medication Adherence in Patients With Type 2 Diabetes Across Different Health Literacy Levels: An Explanatory Sequential Mixed Methods Study

**DOI:** 10.3389/fphar.2021.745749

**Published:** 2021-10-06

**Authors:** Yen-Ming Huang, Olayinka O. Shiyanbola

**Affiliations:** ^1^ Graduate Institute of Clinical Pharmacy, College of Medicine, National Taiwan University, Taipei City, Taiwan; ^2^ Division of Social and Administrative Sciences, School of Pharmacy, University of Wisconsin-Madison, Madison, WI, United States

**Keywords:** diabete, facilitator, barrier, health literacy, medication adherence, mixed methods

## Abstract

Type 2 diabetes (T2D) incurs tremendous healthcare costs associated with various complications due to poor blood sugar control. Medication adherence, which is correlated with patients’ health literacy, should be consistently practiced to achieve optimal control of blood sugar. A comprehensive understanding of specific communication and psychosocial factors related to medication-taking behaviors across different levels of health literacy among people with T2D will guide the development of effective interventions and strategies to enhance medication adherence. To understand barriers and facilitators to medication adherence in people with T2D across different health literacy levels, the Health Literacy Pathway Model was used to identify the psychosocial and communication factors that may influence medication adherence. This mixed methods study used an explanatory sequential design, including a quantitative survey followed by qualitative semi-structured interviews. Two hundred and five participants completed the survey questionnaire, and 23 participants completed semi-structured interviews. Confirmed by quantitative and qualitative data, having stronger self-efficacy and fewer concerns about medications, as well as experiencing fewer perceived barriers to medication-taking, are necessary for better medication adherence among those with low adherence. Our findings will be useful to tailor interventions for diabetes care through addressing concerns among low-adherent patients with low health literacy and emphasizing self-efficacy and perceived barriers to medication adherence among all low-adherent patients with T2D.

## Introduction

Diabetes has been the 7th leading cause of death in the United States for decades, affecting 10.5% of the United States adult population in 2018 (Centers for Disease Control and Prevention, 2020). The management of diabetes incurs remarkable economic burden because of its complications and adverse outcomes largely attributed to poor blood sugar control (Rhee et al., 2005). One of the most effective approaches to attaining a desired glycemic goal is taking medications (Zullig et al., 2015). The World Health Organization (WHO) emphasizes medication adherence - the extent to which individuals take medications as prescribed with respect to the timing, dose, and frequency during the prescribed length of time (Osterberg and Blaschke, 2005) - is the primary attribute to optimal patient health outcomes (Sabaté, 2003). Compared with non-adherent patients, adherent patients have a lower healthcare cost, including fewer hospitalizations and emergency department visits, reduced costs of acute and outpatient care, and better health outcomes (Krass et al., 2015). However, fewer than a third of individuals with T2D in developed countries are considered adherent patients (Gottlieb, 2000), one of the lowest rates among people with chronic illnesses (Donnan et al., 2002). Consequently, increasing the effectiveness of adherence interventions will have a far greater impact on public health than any improvement in specific medical treatments (Sabaté, 2003).

Given that medication non-adherence is an obstacle to diabetes management, understanding the reasons why patients do not take diabetes medications as prescribed is a logical first step to an effective intervention (Mayberry et al., 2013). Research using large claim databases has identified key demographic factors that are associated with medication non-adherence in T2D, such as younger age, lower education level, and lower income (Kirkman et al., 2015). In addition, patients’ psychosocial factors (e.g., beliefs in medication, illness perceptions, and social support) and patient-provider relationships are prominent factors that healthcare professionals can effectively address and change in clinical practice to improve patients’ medication adherence (DeWalt et al., 2007; Gellad et al., 2011; Shiyanbola et al., 2018). Over the past decades, a range of proposed interventions have been implemented to improve patients’ adherence to diabetes medications, including easier access to medication, provision of printed and digital materials, and providing medication reminders (Presley et al., 2019). Despite the advancement of tailored interventions, increase in medication adherence rates (from 4 to 11% of the population) is limited and smaller than expected (Peterson et al., 2003). To date, interventions aimed at improving medication adherence have only been partially successful, in part due to failing to address other salient factors (e.g., health literacy, self-efficacy of medication use, and beliefs in medicine) in intervention programs (Garcia-Perez et al., 2013; Costa et al., 2015). One such factor is health literacy, which indicates an individual’s skills and ability to obtain, process, and use information and services to act effectively in the healthcare environment and make appropriate health-related decisions (Al Sayah et al., 2013; Haun et al., 2014).

Nevertheless, current empirical research has not yet systematically investigated what psychosocial factors healthcare professionals should address, when communicating with patients, given the specific health literacy level of each patient. Psychosocial factors encompass a range of factors related to individuals’ psychological state and social environment and potentially influence their behaviors and health (Upton, 2013). Beliefs in medicine, an example of a psychosocial factor, shapes patients’ perceptions of personal need for medication use and then influence the way patients take their medications (Horne and Weinman, 1999; Huang et al., 2020a). Existing quantitative research on diabetes care has recognized health literacy as a salient predictor of medication adherence via its direct impact on psychosocial factors (e.g., beliefs, self-efficacy, and social support), moderated by patient-provider communication, but the correlational nature of these studies offers limited knowledge of successful strategies to improve patients’ medication adherence (Bailey et al., 2014). For example, people with better numerical operation skills may have stronger confidence when interpreting the reading of a glucose meter, so might be better at taking their diabetes medications as prescribed (Huang et al., 2018a). Current qualitative studies, on the other hand, have explored patient perspectives on factors that may hinder (e.g., concerns about side effects of medications) or facilitate (e.g., strengthened self-efficacy for medication use) medication adherence (Jeragh-Alhaddad et al., 2015; Kvarnstrom et al., 2018; Laranjo et al., 2015). However, researchers tended to neglect patients’ level of health literacy, the antecedent of psychosocial barriers and facilitators. As a result, providers were not able to offer tailored intervention programs, compromising the effect of such programs on medication adherence when the information was beyond patients’ level of health literacy (Gellad et al., 2011; Kvarnstrom et al., 2018; Laranjo et al., 2015; Sohal et al., 2015). That is, studies using qualitative or quantitative approaches alone may provide limited insight into the development of successful clinical interventions. To revamp current intervention practices for diabetes care, following comprehensive theoretical models and integrating quantitative and qualitative research into a mixed methods approach can effectively enrich our knowledge of non-adherence among people with T2D (Costa et al., 2015).

### Theoretical Framework

The Health Literacy Pathway (HLP) Model shows a promising way to better contextualize predictors of medication adherence among patients with different levels of health literacy (Paasche-Orlow and Wolf, 2007). The HLP model describes how individuals’ psychosocial factors and their ability to communicate with healthcare professionals may influence health behaviors (e.g., medication adherence) (Paasche-Orlow and Wolf, 2007). In the HLP model, health literacy is theorized to influence individuals’ self-efficacy, motivation, perceived barriers, and their communication with providers. These factors in turn contribute to how individuals take their medications. The theory sheds light on the extent to which individuals’ communication and psychosocial factors (e.g., beliefs, self-efficacy, motivation, and perceived barriers) have a direct linkage to their health behaviors. Furthermore, the HLP model advocates the investigation of these individual-level factors within contexts that could be incorporated into clinical practice. As a result, healthcare providers are able to tailor their communication contents to patients’ need to improve medication adherence by strengthening their self-efficacy, enhancing their motivations, and addressing their barriers to medication use.

In addition, the mixed methods approach is deemed essential to enrich existing knowledge of medication adherence through mapping qualitative onto quantitative results under the framework of the HLP model (Curry et al., 2009). Nonetheless, to date, only a handful of research has adopted mixed methods to understand patient factors related to medication adherence of people with T2D (Aloudah et al., 2018; Rao et al., 2020). Notable exceptions include Aloudah et al., (2018) who used an explanatory sequential mixed methods design to assess adherence to oral diabetes medications among people with T2D and explore factors correlated with adherence behaviors. Their results underlined specific behavioral factors (e.g., complex scheduling medication taking) and social influences (e.g., lack of family support) of medication non-adherence, providing deeper insights into intervention development (Aloudah et al., 2018). Rao et al. (2020) conducted a longitudinal evaluation of changes in medication adherence and determined barriers and facilitators of medication adherence among Blacks with T2D through an explanatory sequential mixed methods design. Their findings suggested that specific beliefs in diabetes medication be addressed to improve medication adherence of Blacks with T2D (Rao et al., 2020). As such, mixed methods can provide a deeper insight into the questions of interest and yield complete evidence through mutually complementing results to depict complex phenomena (Creswell et al., 2004; Creswell and Clark, 2017).

A mixed methods approach fits this study’s research questions well. For one, medication non-adherence is multifaceted, demanding nuanced ways to thoroughly describe the behavior. For the other, merging the findings of quantitative and qualitative approaches could enrich the interpretation of one type of results by being informed from another type. A theory-driven, mixed methods approach is thus expected to elicit a better understanding of intricate ways in which healthcare practice (e.g., communication between healthcare providers and patients) and psychosocial factors contribute to medication adherence in light of the level of health literacy (Hadi et al., 2013, 2014). Findings from this study are expected to guide the development of effective interventions and customized strategies to enhance patients’ medication adherence and improve their diabetes outcomes in the long term.

### Aim

Our quantitative research question aimed to examine whether the barriers and facilitators associated with medication adherence differed among people with T2D across different levels of health literacy. The qualitative research question was to explore patients’ perceptions of the barriers and facilitators of medication adherence across different health literacy levels. The mixed methods question sought to understand how the qualitative data reported about the barriers and facilitators of medication adherence helped explain the quantitative results reported on the survey.

## Methods

In the present study, we focused on medication adherence instead of persistence, which describes the degree to which an individual continues taking medications for the prescribed duration (Cramer et al., 2008) A facilitator was defined as any situational or individual factor identified by study participants that improves adherence to the diabetes medications as prescribed. A barrier was any situational or individual factor identified by study participants that hinders adherence to the diabetes medications as prescribed.

### Study Design

**FIGURE 1:**
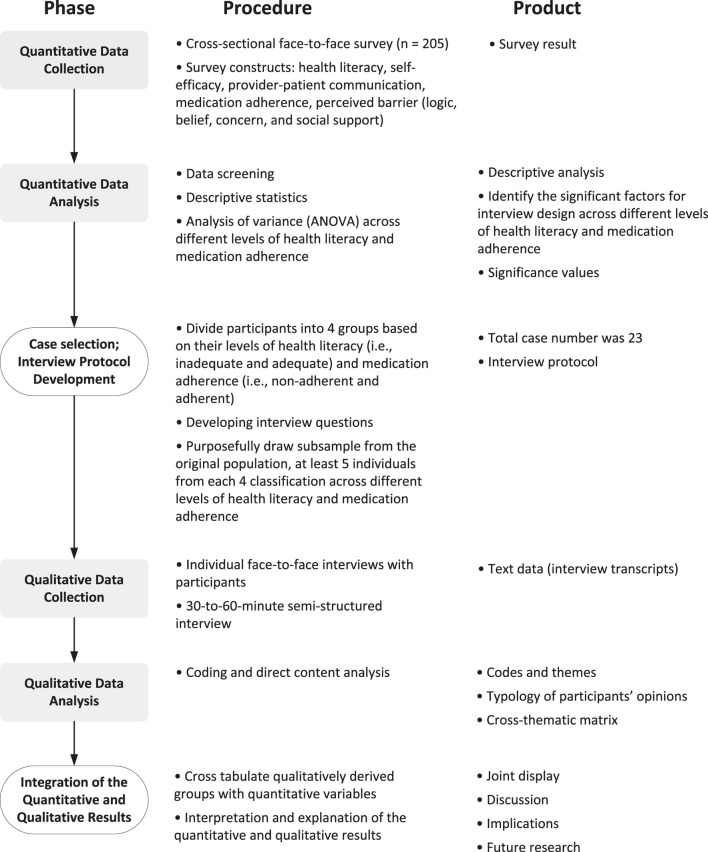
Explanatory sequential mixed methods design of the exploration of barriers and facilitators of medication adherence.

This cross-sectional study utilized an explanatory sequential mixed methods design which comprised a quantitative and a subsequent qualitative phase (Figure 1) (Ivankova et al., 2006). This design involved collecting and analyzing the quantitative data first and then exploring the quantitative results with an in-depth qualitative data collection and analysis (Harrison et al., 2020). A mixed methods design can be useful because it enables researchers to use qualitative data and its analysis to further understand the statistical results by exploring the participants’ view in a greater depth (Johnson and Schoonenboom, 2016).

First, at the quantitative phase of the study, a survey was administered at a family clinic, located in a Midwestern state in the United States, to examine the barriers and facilitators associated with medication adherence (Huang et al., 2020b). Second, the qualitative phase consisted of one-on-one interviews, also taking place at the same family clinic, as a follow-up to the survey results to explore the barriers and facilitators that contribute to medication adherence (Huang et al., 2020a). Lastly, the results collected from both quantitative and qualitative phases were integrated to explore how the qualitative findings underpinned the quantitative results. In both phases, the survey and interviews were conducted via a face-to-face approach from November 2018 to May 2019.

### Recruitment and Eligibility Criteria

A patient list of potential participants was gathered from a family medical center in a Midwestern state by one of the study researchers. This list was accessed from the electronic health record (EHR) database to identify patients that met the inclusion criteria. Study participants were eligible if they were at least 20 years of age, diagnosed with T2D, presently being prescribed to take at least one diabetes medication by mouth daily, and able to read and speak in English. Individuals who were younger than 20 years old, too ill to participate in the survey, presently not being prescribed any oral medications for diabetes management, or unable to understand English were excluded. Patients with diabetes were identified based on an International Classification of Diseases, Tenth Revision, Clinical Modification diagnosis code of E11.XXX. Each participant provided a signed informed consent to take part in the study.

### Definition of Variables

**FIGURE 2:**
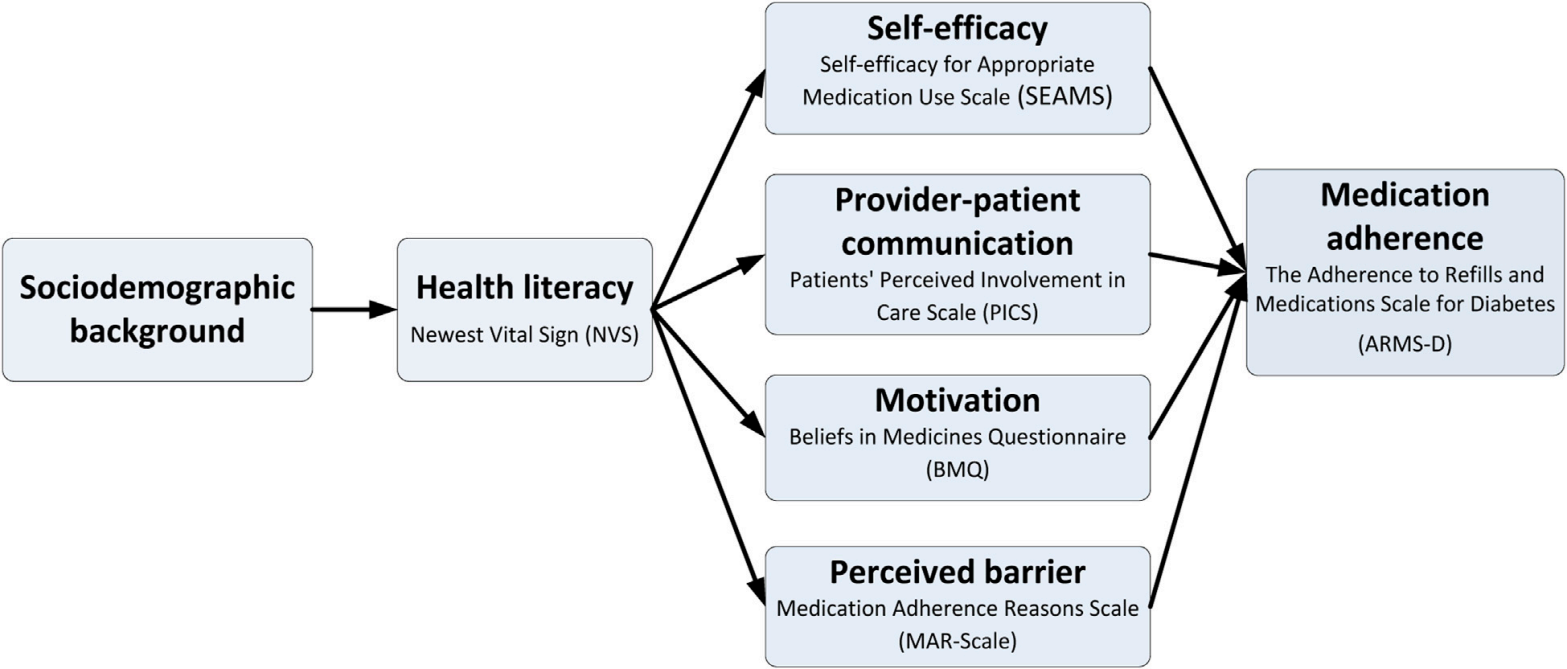
Conceptual framework of medication adherence and its associated factors based on the Health Literacy Pathway Model.

We applied the HLP model to identify the salient constructs associated with diabetes medication adherence and health literacy (Paasche-Orlow and Wolf, 2007). Following the HLP model, this study focused on factors that are associated with both health literacy and medication adherence at individual and interpersonal levels (Figure 2). The key factors, based on the HLP framework, included 1) self-efficacy, 2) motivation, 3) perceived barriers, and 4) provider-patient communication. In this study, self-efficacy was defined as patients’ beliefs in their ability to take medications under prescribers’ instructions. Motivation referred to patients’ beliefs about medications which drove them to adhere (or not) to their medications. Perceived barriers were explained by subjective reasons for medication non-adherence. Provider-patient communication represented communication experiences based on patients’ perceptions of past clinic visits.

### Quantitative Phase

#### Data Collection

An 85-item survey was administrated via a face-to-face approach over 4 months. Participants were recruited from the identified patient list; every patient on the list was invited. At the research site, we provided the potential participants with the study information sheet and allowed them about 5–10 min to review it. The researchers engaged participants in the recruitment process and discussed the study in detail. Interested and eligible patients were taken to a private area to complete the informed consent form and then the survey, which took participants 17 min on average to complete (range: 7–28 min). Each participant was compensated $20 cash for their time.

#### Measurements

##### Sociodemographic and Clinical Characteristics

We collected sociodemographic information (i.e., age, gender, sex, the highest level of education, and the annual household income) and clinical characteristics (i.e., the type of diabetes medications, duration of diagnosis of diabetes mellitus, and HbA1c). HbA1c values were abstracted from electronic medical records using the most recent value for each participant within the prior 6 months.

##### Health Literacy

Health literacy was measured by the 6-item Newest Vital Sign (NVS), which tests the respondent’s literacy, comprehension, application/function, evaluation, and numeracy skills (Haun et al., 2014; Huang, et al., 2018b; Weiss et al., 2005). In the NVS, an interviewer asked six questions relating to information contained in an ice cream nutritional label (Weiss et al., 2005). For example, people were asked “If you eat the entire container, how many calories will you eat?” to evaluate their ability of numerical operation. The total score of the NVS ranged from 0 to 6, and higher scores indicated better health literacy (Weiss et al., 2005). In this study, those with scores greater than 3 were regarded as having adequate health literacy, and the rest of the participants were deemed to have inadequate health literacy (Weiss et al., 2005).

##### Self-Efficacy for Medication Use

Self-efficacy for medication use was assessed using the 13-item Self-efficacy for Appropriate Medication Use Scale (SEAMS) (Risser et al., 2007). Participants were asked to indicate, under a number of different circumstances, their level of confidence about taking medications correctly. Each item was evaluated using a 3-point Likert-type scale (1 = not confident, 2 = somewhat confident, 3 = very confident). The sum of SEAMS ranged from 13 to 39. Higher scores reflect the respondents have higher self-efficacy of taking their medications as prescribed (Risser et al., 2007).

##### Motivation

Participants’ motivation for medication adherence was measured using the 10-item Beliefs about Medicines Questionnaire-Specific (BMQ-Specific), which consists of two 5-item subscales: necessity beliefs and concern beliefs (Horne et al., 1999). Each item was measured on 5-point Likert-type scales with ‘strongly disagree (score = 1)’ to ‘strongly agree (score = 5)’ response options. The scores summed for each subscale ranged from 5 to 25, with a higher score meaning stronger necessity or concern beliefs about the medication prescribed for personal use (Horne et al., 1999).

##### Patient-Provider Communication

Patient-provider communication was assessed using the 13-item Perceived Involvement in Care Scale (PICS) (Lerman et al., 1990). The PICS evaluats doctor-patient interactions across three relatively distinct factors, including doctor facilitation of patient involvement, level of information exchange, and patient participation in decision making. In total, respondents answered 13 dichotomous items (0 = no, 1 = yes). Items scores were summed to generate a total score ranging from 0 to 13, and higher scores suggest a greater degree of shared decision making for disease self-management (Lerman et al., 1990).

##### Perceived Barrier

Perceived barriers to medication adherence was evaluated using the 20-item Medication Adherence Reasons Scale (MAR-Scale), which measures patients’ perceptions of medication adherence (Unni et al., 2014). Participants were asked to answer the number of days they did not adhere to their medications over the past week, according to the various reasons listed for non-adherence. Each item was anchored on an 8-point scale ranging from 0 day to 7 days (Unni et al., 2014). In this study, the barrier score was calculated by counting the number of barrier items that participants reported (i.e., scored > 1) over the past 7 days. As a result, the total score of the perceived barriers ranged from 0–19, with a higher score meaning more barriers to taking diabetes medications as prescribed.

##### Medication Adherence

Medication adherence was evaluated using the 11-item Adherence to Refills and Medications Scale for Diabetes (ARMS-D) (Mayberry et al., 2013). The ARMS-D asks participants about their daily experiences regarding self-administration or refill of their diabetes medications in the past 3 months. These 11 items comprise 2 subscales, a 7-item medication-taking subscale and a 4-item medication refill subscale. Each item was measured on a 4-point Likert-type scale (1 = none of the time, 2 = some of the time, 3 = most of the time, 4 = all of the time). The responses were reverse-scored, such that higher scores indicated better medication adherence. A maximum sub-score of 28 and 16 indicated a high adherence to taking and refilling prescribed medications, respectively (Mayberry et al., 2013). Based on the score of medication-taking subscale of the ARMS-D, participants were classified as high-adherent when they scored 28 and low-adherent when they scored less than 28.

#### Data Analysis

Participants were divided into four groups based on their scores of the NVS and ARMS-D: 1) inadequate health literacy and high medication adherence, 2) adequate health literacy and high medication adherence, 3) inadequate health literacy and low medication adherence, or 4) adequate health literacy and low medication adherence. We compared the differences in targeted psychosocial and communication factors (i.e., self-efficacy, patient-provider communication, motivation, and perceived barriers) between the four groups. One-way analysis of variance (ANOVA) was used to compare the mean scores of these factors across the four different groups. Post hoc analyses were conducted using Dunnett’s C post hoc criterion for significance. All statistical analyses were carried out using SPSS version 26, with the statistical significance level at a two-sided *p* < 0.05.

### Qualitative Phase

#### Data Collection

A purposive maximum variation sampling was applied to recruit participants for interviews to maximize the depth and richness of data. Every participant who completed the survey and consented to be interviewed was contacted and invited in the qualitative phase. Those who were successfully contacted were all interviewed. Individuals who completed the survey were eligible for the qualitative phase. For those who agreed and consented to participate, the interview was scheduled 1 hour before the participant’s next clinic visit or any time convenient for the participant. An in-depth one-on-one, face-to-face interview was conducted to understand participants’ experiences of taking medications for diabetes management. Participants took part in semi-structured interviews in private rooms on the research site, and the researcher audio-recorded the interview and took brief notes during each 30–60-min interview. Interviews began with a general question that facilitated conversation between the researcher and interviewees, and sequent questions focused on participant’s experiences of taking diabetes medications. During the interviews, the researcher did not interrupt but used cue phrases (actual words spoken by the participant) to elicit more detailed narratives (Savin-Baden and Major, 2013). To generate richer information, the interviewees were asked probing questions and allowed to add any information they deemed to fit the questions. The median length of interview was 46 min (range: 22–70 min). Each participant received $20 cash upon completion of the interview.

#### Qualitative Interview Tool

An interview protocol was created and served as a guide in each interview to prevent the researcher from uncontrolled deviation from the research topic. The interview questions (Supplementary Appendix S1) were developed based on the HLP framework and revised by three professionals with expertise in health literacy, qualitative methodology, and medication adherence to ensure the questions were relevant to the research aims.

#### Data Analysis

Each audio-recorded interview was transcribed verbatim, and the researcher verified each interview transcript against its corresponding audio recording. Direct content analysis, guided by the HLP model, was used for interview analysis. Direct content analysis is usually appropriate when existing theory or research literature can guide and identify key concepts or variables of interest (Hsieh and Shannon, 2005). Based on Elo and Kyngäs’s (2008) philosophy, researchers analyze the data operationalized on the basis of previous knowledge rather than the naive perspective. The advantage of the direct content analysis is that existing theory can be supported and extended (Hsieh and Shannon, 2005). Researchers are thus able to compare new findings with previous literature to extend and add to existing theory to enrich the knowledge of topics of interest.

The analysis included the following steps: 1) initially reading the transcripts three times to achieve immersion; 2) reading the data line by line to capture key thoughts; 3) coding and organizing the themes that mapped onto the constructs informed by the HLP model (Miles et al., 2018). The coding process consisted of two stages. First, the researcher assigned the data chunks to generate main codes, and then used both descriptive coding and *in vivo* coding (Miles et al., 2018). In the first-cycle coding, we initially summarized the segments of data and condensed large amounts of data into a smaller number of analytic units. In the second stage, pattern coding was used to group these units into a smaller number of categories or themes in line with the constructs from the HLP model. We used investigator triangulation to develop an overall coding taxonomy and ensure the trustworthiness of the results (Thurmond, 2001; Archibald, 2016). For all transcripts, two research members independently coded the transcripts and came to consensus on each code and the interpretation to ensure the findings were grounded in the texts. MAXQDA 12 was used to organize and categorize the themes.

### Integration of the Quantitative and the Qualitative Data Sets

Integration was implemented at three levels, including the design, methods, and interpretation/reporting levels, respectively (Fetters et al., 2013). We conducted an explanatory sequential design to implement integration at the design level (Creswell et al., 2004). At the methods level, we performed integration through connecting by linking the quantitative and qualitative approaches through sampling (Fetters et al., 2013) i.e., the interview participants being sampled were from those who completed the survey. Lastly, we implemented integration at the interpretation and reporting level through a joint display (Guetterman et al., 2015). The joint display in an explanatory design was used to integrate the findings from the aforementioned quantitative and qualitative phases so the categories and themes that emerged from the analysis of the interviews were used to explain the results from the survey (Creswell and Clark, 2017). We selected the constructs showing statistical significance and mapped these quantitative results onto the corresponding findings from qualitative interviews. As such, we were able to explore the reasons why participants across the four groups reported different levels in the variables of interest. We organized the findings in integrated results matrices that juxtaposed the findings from the quantitative and qualitative analysis (Fetters et al., 2013). This side-by-side joint display allowed us to compare the findings of different approaches simultaneously through a visual means to draw meta-inferences and new insights beyond the information from the separate quantitative and qualitative results (Guetterman et al., 2015).

## Results

### Quantitative Findings

Two hundred and five (mean age = 60.9 years, SD = 10.2) out of 218 patients who agreed to take part in the study completed the survey, with a response rate of 94.0%. Most of the participants were female (*n* = 116, 56.6%) and non-Hispanic White (*n* = 152, 74.1%); most had at least a college degree (*n* = 128, 62.4%). Forty-two percent (*n* = 88) of the participants took both oral hypoglycemic agents (OHA) and injectable medications for diabetes management, whereas 51.7% (*n* = 117) of the participants only took OHAs. The mean duration of time since diabetes was diagnosed was 8.95 (SD = 6.31, median = 8) years with mean HbA1c level 8.17% (SD = 1.88, median = 7.8%). Supplementary Appendix S2 describes participants’ sociodemographic backgrounds.

The measures of NVS, SEAMS, BMQ-Specific, PICS, MARS-Scale, and ARMS-D yielded high internal consistency with Cronbach’s alpha values of 0.76, 0.91, 0.72, 0.76, 0.82, and 0.85, respectively. On average, the NVS score of all participants was 4.26 (SD = 1.73), and 72.7% (*n* = 149) and 27.3% (*n* = 56) of the participants had adequate or inadequate health literacy, respectively. The mean score of the self-reported medication adherence, one of the subdomains of ARMS-D, was 26.02 (SD = 2.44); 56.6% (*n* = 116) and 43.4% (*n* = 89) of the participants were considered of low and high medication adherence, respectively. Better medication adherence showed a positive association with HbA1c (*r* = 0.324; *p* < 0.001), indicating participants with higher adherence to diabetes medications were more likely to have better control of their blood sugar than those with low-adherence. Based on participants’ self-reported health literacy and medication adherence, they were categorized into four distinct clusters, including 1) inadequate health literacy and high medication adherence (*n* = 23; 11.2%), 2) inadequate health literacy and low medication adherence (*n* = 33; 16.1%), 3) adequate health literacy and high medication adherence (*n* = 66; 31.2%), or 4) adequate health literacy and low medication adherence (*n* = 83; 40.5%).

Supplementary Appendix S3 demonstrates the differences of each cluster in psychosocial and communication factors. Significant differences were found between four clusters based on self-efficacy (*p* < 0.001), concern beliefs (*p* = 0.007), and perceived barriers (*p* < 0.001). Participants in the clusters with high medication adherence reported higher self-efficacy and fewer perceived barriers to medication-taking than those in clusters with low-adherence, regardless of their health literacy levels. Compared with participants with adequate health literacy and high medication adherence, more concerns about medications (i.e., concern beliefs) were raised by participants from the cluster of low medication adherence and inadequate health literacy. Therefore, higher self-efficacy and lower perceived barriers were regarded as facilitators of medication adherence among low-adherent clusters regardless of their health literacy. In contrast, concern beliefs were deemed as a barrier to medication adherence in the cluster of low medication adherence and inadequate health literacy.

### Qualitative Findings

Twenty-three participants were recruited for qualitative interviews, including individuals with 1) inadequate health literacy and high medication adherence (*n* = 5), 2) adequate health literacy and high medication adherence (*n* = 5), 3) inadequate health literacy and low medication adherence (*n* = 6), or 4) adequate health literacy and low medication adherence (*n* = 7). Since more diverse opinions emerged during the interviews of participants with low medication adherence, more participants in the low-adherent clusters were recruited to capture richer information of how they managed medications for diabetes care. With no rigid rule related to the sample size for qualitative interviews, a sample size of 15–30 is sufficient for a content analysis approach (Francis et al., 2010). The sample was composed of 12 females (52.2%) and 11 males (47.8%), with the age range of 40–78 years old.

Guided by the HLP model, factors related to barriers and facilitators of medication adherence were coded into four categories: 1) self-efficacy, 2) communication, 3) motivation, and 4) other perceived barriers. The themes identified as barriers or facilitators that may influence medication adherence are explained with direct verbatim quotes for further clarification.

#### Facilitators of Medication Adherence

##### The sense of being able to control diabetes medications enhances self-efficacy of medication use

Most of the participants felt that they were equipped with clear understanding of medication instructions and advanced skill in medication administration, so it was easy to take medications on their own. The feeling of full control of medication administration on their own may have strengthened participants’ self-efficacy of medication use.

“I think it’s just easier to take those medications, because I know what they can, I have a good idea of what they can do, especially the insulin.”

##### Having medication-taking as a part of daily routine supports self-efficacy of medication use

Most of the participants emphasized that turning medication-taking into a habit helped them remember to take medications. They referred to using visual and automated reminders as a facilitator for medication-taking on a regular basis.

“I have a tall dresser in my room where my grandkids can’t get up on it, so it’s the first thing I see because it’s directly across from my eyesight when I wake up in the morning. So it kind of helps me remember to take [my medications].”

##### Patients consider good rapport with providers as a support for diabetes management

Participants indicated that providers’ respect of their opinions in diabetes management empowered them to be more engaged in their diabetes care through starting more conversations and providing rationales for the treatment. The good relationship between patients and providers made patients more informed of their treatment and motivated them to take medications persistently.

“[my doctor] informs me as to the whys and which medication is serving which purpose. When we had a change and had to go into the insulin, when we mutually decided on that, he informed me how that was the better management tool for the diabetes at that point. He just tells me what and why, and it works from there. If I understand what is being done and why it is being done, that helps make it a whole heck of a lot easier too.”

##### Belief in the effectiveness of treatment strengthens the perceived need of diabetes medications

Participants’ perceived necessity of medication forged desirable diabetes management. All participants agreed that medication was essential for diabetes management because it helped regulate blood sugar and prevent diabetes-related complications.

“[Diabetes medications] have been very effective. I was able to get the disease under control very quickly through the use of the medication, obviously, was very reinforcing to me, as far as its effectiveness. So I want to, obviously, continue that good, those good results.”

#### Barriers to Medication Adherence

##### The sense of being overly controlled by diabetes medications decreases self-efficacy of medication use

Low-adherent participants mentioned that they would like to own their diabetes self-management rather than be externally controlled by medications. They were averse to taking medications as prescribed due to perceived overwhelming control by medications and feeling of a lack of autonomy in diabetes management.

“I just be tired of taking medication. Everything that I think I need to do is stuff within myself that I need to decide that I’m going to do, and sometimes I just don’t do that.”

##### Being unable to integrate medication-taking into routine diminishes self-efficacy of medication use

Several low-adherent participants expressed that they had difficulties in taking medications as a part of daily routine. Some of them mentioned that it was hard to take medications at regular time due to some situational influences.

“So I don’t know, just getting into the habit of doing it is tough. And like I said, I think I’m in denial still that I have it, and I don’t want it. It just seems to take a lot of time, and it really doesn’t, but it does. It’s hard for me to get into the routine. So that’s just a bunch of needles and, hmm, just getting it all taken care of, so it’s been hard.”

##### Lack of trust in providers sabotages the relationship between patients and providers

A few low-adherent participants mentioned that a poor patient-provider communication risked their relationship with providers. Some participants felt that their providers did not listen to patients but forced them to follow the provider’s orders instead. As a result, participants thought that their providers did not care about their health and were reluctant to take medications as prescribed.

“I’ve always just kind of felt that my doctor really just didn’t care too much. It’s gotten a little bit better, but he still just seems like detached. He doesn’t have any vested interest in my health, so why should I [take medications]?”

##### Concerns about medication safety impedes motivation for taking medications

Participants mentioned that they were concerned about side effects of the medication they used. Some participants shared that they formed their concerns based on their past experiences with a few side effects from the medication they used.

“My stomach got so upset all the time. I got the diarrhea, and I just felt better after I cut it off, and I left it at one…So, yes, I cut it off myself without doctor’s permission.”

##### Confusion about the role of medications hinders the motivation for medication-taking

Lacking an awareness of the role of medications was identified as a barrier to medication adherence. Participants with low medication adherence doubted the effectiveness of medication. Those with inadequate health literacy mentioned that they were short of knowledge of diabetes itself and confused with information from healthcare professionals. While those with adequate health literacy were able to search relevant information at hand, they also had difficulties handling conflicting information from their self-searching and healthcare providers.

“When I was on [the medication] for like two years, and I was healthy and everything else. Four to six months then, and then it all went bad. Everything shot, the bad nerve pain in my hands and my feet. Now it’s all coming back again, and that’s what’s scaring me. I don’t understand what, why the diabetes does that. I don’t know a lot about diabetes though.”

##### Other perceived barriers hindering adherence to medication

Some participants with low mediation adherence described distress (e.g., seasonal depression) hampered their medication-taking. Some participants indicated limited access to medications (e.g., cost and insurance) confined their ability to take medications as prescribed. In addition, some participants pointed out a complex diabetes regimen was an obstacle to medication adherence.

### Mixed Methods Findings

Findings from the quantitative results showed significant differences in three constructs between participants from varying clusters: self-efficacy of medication adherence, concerns about medication, and perceived barriers to medication use. Themes from the qualitative data analysis corresponding to quantitative results were subsequently presented using an integrated approach, which drew on any data of relevance for the research aims. The joint display augmented these key quantitative findings with qualitative interviews to explain the nuances of these differences (Table 1, Table 2 and Table 3).

**TABLE 1:** Integrated mixed methods results matrix of self-efficacy of medication use

Quantitative data summary	Exemplar quote	Qualitative data summary	Meta-inference (Merging/Integrating results)
• Participants with high MA self-reported significantly stronger self-efficacy than those with low MA regardless of their HL.	I’m always confident about taking my meds. I don’t find anything hard about it. […] I know I can take it on my own. (P5)I just be tired of taking medication. Everything that I think I need to do is stuff within myself that I need to decide that I’m going to do, and sometimes I just don’t do that. (P18)	• A sense of being able to control medications for diabetes management on one’s own fostered self-efficacy, but a feeling of being overly controlled by medications did not.	• Confirmed by quantitative and qualitative data, holding stronger self-efficacy was necessary for better MA; participants’ level of health literacy determined their ability to use handy helpful resources to link medication-taking to daily routine.
	I link my routine to my medicine. I just go to my satchel. […] I take out my pill bottle, and I put, and so I always know where it is. It’s easy to find, so it’s easy to manage. (P1)My medication is set up in a med box provided by the university pharmacy so my medication is distributed to me in little packets, four packets a day. […] It’s very easy for me to follow that. (P9)	• Incorporating medication-taking with daily routine helped develop confidence in medication-taking, and those with adequate HL used more strategies to link medication-taking to routine.	
	Sometimes it’s inconvenient, and I haven’t had a chance to eat to take it. You get busy, or you just don’t feel like it’s working. I’m not confident it’s working period. (P16)Sometimes I’m out of town, and I forgot to take [my medication]. I don’t take it is when I’m out of town, or I just ain’t got, want to eat that day. (P13)	• Perceived situational influences impeded participants’ confidence in MA, and those with inadequate HL perceived more situational influences than those with adequate HL.	

Note: HL, health literacy; MA, medication adherence

**TABLE 2:** Integrated mixed methods results matrix of concerns about medication

Quantitative data summary	Exemplar quote	Qualitative data summary	Meta-inference (Merging/Integrating results)
• Under the same level of MA, concerns about medication did not statistically significantly differ across clusters.• The low-adherent cluster with inadequate HL had the highest concerns about medications.	It wasn’t one time I pooped out the pill, it was like every day for a week I kept pooping this pill out. That was like my body was rejecting that pill, so that drew a concern to me. (P5)I’ve wondered how, is this here something that I have to take for the rest of my life? […] Will my body get immune to these pills after taking them for so long? I won’t know until I talk to my doctor. Or I can just go online and look it up. (P6)	• High-adherent participants had concerns, which was alleviated by getting more support from providers or online resources.	• Confirmed by quantitative and qualitative data, concerns existed in all clusters, and participants’ health literacy determined how they used available resources to cope with their concerns.• Low-adherent participants had more problems coping with their concerns. In particular, those with inadequate HL tended to skip their medications due to unsolved concerns.
	I started gaining weight again, and then it all went bad. And then I had to go back on my meds and increase the milligrams of both that I was taking. And then everything shot, the bad nerve pain in my hands and my feet. Now it’s all coming back again, and that’s what’s scaring me. And I don’t understand what, why the diabetes does that. I don’t know a lot about diabetes though. That’s why you sometimes don’t want to take it. Or is it going to work? Is this the right medication for me? (P16) [The doctor] had me taking two of them pills. I cut back to one, it just, got sick off of them. My stomach got so upset all the time. I got the diarrhea, and I just felt better after I cut it off, and I left it at one. […] I did cut it off by myself without the doctors being told. (P12)	• Low-adherent participants could not resolve their concerns, and that may have influenced their decision-making regarding medication use.	

Note: HL, health literacy; MA, medication adherence

**TABLE 3:** Integrated mixed methods results matrix of perceived barriers to medication adherence

Quantitative data summary	Exemplar quote	Qualitative data summary	Meta-inference (Merging/Integrating results)
• There was a significant difference in perceived barriers to medication-taking as prescribed between the clusters of high and low MA.• Low-adherent clusters reported more perceived barriers to medication-taking over the past 7 days, but high-adherent clusters did not.	Sometimes the injectable pens, they don’t push as easy as some, some others do. I have some problems with my hands, and so sometimes it’s hard for me to maneuver the pen with still having the needle, injected in my stomach. (P1)For me, the hardest part, although it’s not a large deterrent, is the physical size of the pills, physically swallowing them. Because I have to take four, and they are very big. (P7)	• High-adherent clusters tended to keep taking medications as prescribed despite some perceived barriers to medication adherence.	• Confirmed by quantitative and qualitative data, participants with fewer perceived barriers to medication-taking tended to have better MA.• High-adherent participants tended to find a way to handle possible barriers to medication use and keep taking medications, but low-adherent participants did not.
	It’s just sometimes it was inconvenient or just got too tired and forgot to take them. (P12)Other situations in which I’ve not taken it when I’m supposed to is when I’ve had a problem affording the insulin and maybe had to go without it for a few days because I haven’t gotten enough money to buy the insulin. Or I know I’m going to be running out of insulin in the near future, and so I take less of a dosage than I should. (P23)	• Low-adherent clusters perceived more barriers to medication adherence and did not take their medication as prescribed.	

Note: HL, health literacy; MA, medication adherence

#### Self-Efficacy Impacts Medication Adherence

The quantitative data suggested that stronger self-efficacy of medication use was positively associated with medication adherence. (Table 1). Analysis of the interviews indicated that participants felt confident in their medication use as they could control their medications for diabetes management on their own. However, participants with low adherence were less confident in taking medications as prescribed. Meanwhile, they also expressed a fear of losing their control over their body to medications. In addition, incorporating medication-taking with daily routine helped develop self-efficacy of medication use, and those with adequate health literacy were prone to use multiple strategies to incorporate medication-taking into their routine. More perceived situational (e.g., being away from home) influences impeded participants’ confidence in medication-taking, and those with inadequate health literacy perceived more situational influences compared with those with adequate health literacy (Table 1).

As a result, having stronger self-efficacy was necessary for better medication adherence, and linking medication-taking to daily routine was regarded as an imperative approach to foster self-efficacy in medication use. Being able to employ available resources to incorporate medication-taking into daily routine highly relied on health literacy. Of note, participants tended to stop taking medications when they could not handle the inconvenience from perceived situational influences (e.g., busy schedule), which occurred more often among those with inadequate health literacy (Table 1).

#### Concerns Matter for Medication Adherence

The quantitative data indicated that concerns about medication were a barrier to medication adherence. Concerns about medication did not differ across clusters of the same level of medication adherence. However, the cluster of low-adherence and inadequate health literacy reported the highest concerns about medications which was significantly higher than the concerns of the high-adherent cluster with adequate health literacy (Table 2). The qualitative analysis further helped explain the quantitative findings as it showed that high-adherent participants received more support from providers or online resources to tackle their concerns with medication. Conversely, low-adherent participants had difficulties resolving the concerns with their medications. These difficulties in turn influenced their decision-making regarding medication use. Confirmed by quantitative and qualitative data, concerns with medications existed in all clusters, but concerns tended to impede medication-taking among the low-adherent participants with inadequate health literacy.

#### Perceived Barriers to Medication Adherence Need to Be Solved

Analysis of the quantitative data showed that participants with low adherence reported more perceived barriers to medication-taking than high-adherent participants over 7 days. The qualitative results demonstrated that high-adherent clusters kept taking their medications as prescribed despite the barriers to medication adherence. In contrast, low-adherent clusters perceived more barriers to medication adherence and tended not to take their medication as prescribed. Hence, the integrated analysis concluded that participants who perceived fewer barriers to medication-taking inclined to have better medication adherence, regardless of their health literacy (Table 3).

## Discussion

Findings from this mixed methods study support the application of the HLP model to describe psychosocial and communication factors related to medication adherence among people with T2D across different health literacy levels. In doing so, we identified and addressed patients’ barriers and facilitators of medication adherence using theory-driven methods. Among people with T2D, stronger self-efficacy of medication use, and good patient-provider communication facilitate patients’ adherence to their medications. However, more perceived barriers to medication use and concerns about medications keep patients from taking medications as prescribed. Individuals’ health literacy determines their ability to make use of resources to cope with challenges of medication adherence.

### Implications for Healthcare Professionals

The success of diabetes management therapy partly relies on patients’ self-efficacy and motivation, patient-provider communication, and medication adherence (Klinovszky et al., 2019). Healthcare professionals can initiate conversations with patients during clinic visit and check these psychosocial factors to further understand the patient’s barriers to medication use. For example, motivational interviews can help patients resolve ambivalent feelings and insecurities and motivate them to change their behaviors (Moran et al., 2008).

There is no single strategy to overcome all the barriers to medication adherence at once due to its multidimensional nature; therefore, it is essential that interventions address multiple factors in order to improve and sustain adherence (Capoccia et al., 2016; Shiyanbola et al., 2019). A comprehensive intervention that integrates health literacy with psychosocial components may be a more effective strategy to improve medication adherence than an isolated approach (Zhang et al., 2014). Using plain language with understandable materials is a steppingstone to improving health literacy. Helping patients make use of existing resources and addressing their psychosocial factors related to medication use are further steps to improve their adherence to diabetes medications.

Previous research demonstrated that addressing diabetes threats and perceptions of adherence can improve patients’ acceptance and adherence to the treatment (Fall et al., 2013). Aligned with the present study findings and prior literature, open conversations between patients and physicians and encouraging patients to discuss their concerns with medication (e.g., side effects, long-term risks) with physicians may help inform better decisions regarding medication-taking. Misinformation is rampant in diabetes care; directing patients to credible information outlets (e.g., providing a list of reliable and trusted websites, public media, and hotline phone numbers) can help them self-educate on how to make appropriate decisions when healthcare professionals are unavailable. Engaging patients in peer interactions to gain more support from their peers with the same disease management experience will provide benefits to a decision regarding medication use (Moskowitz et al., 2013; Shiyanbola et al., 2021). The use of phone and telehealth interventions and integrative health coaching can be considered to overcome the problems of long distance and the understaffed facility (Capoccia et al., 2016).

Making medication-taking a daily routine is an imperative component to foster patients’ self-efficacy of medication use. Phillips et al. (2016) suggested that habit-based interventions be adopted to improve medication nonadherence using multiple cues for medication adherence. Pharmacists can provide strategies of adherence support to non-adherent patients, including refill reminder calls and using a pillbox or blister packaging (Capoccia et al., 2016). For low-adherent patients, the model of mHealth has been advocated to improve medication adherence, particularly when providers are not available (Fitzpatrick et al., 2019; Park et al., 2019). With the integration of mHealth into diabetes care, patients are able to access accurate information regarding diabetes care, receive reminders to take medications, and have questions answered anytime on their electronic devices (Park et al., 2019). These advantages directly answer the major concerns that participants in the present study have voiced in terms of common barriers to medication adherence. To make the mHealth better serve patients, based on the responses from the participants in this study, mobile apps should provide timely and sufficient information on the effects and side effects of the medication in layman’s term to resolve concerns. Flexible app-based modules to set reminders to help build routines and habits, and proper feedback and reinforcement may improve patients’ self-efficacy.

### Limitations

The interpretation of the findings should be viewed considering the following limitations. First, the study was not an experimental design and thus did not lead to causal conclusions. We were unable to ascertain participants’ continual medication adherence. Prospective research is needed to determine the effects of these psychosocial and communication factors on medication adherence over time to understand the long-term effect of the factors of interest. Second, diabetes medication adherence was assessed based on a self-reported measure rather than using objective measures such as pill count or pharmacy refill information. Self-reported data are prone to systematic biases, including patients’ difficulties in critical self-assessment, recall biases, and social desirability bias (Althubaiti, 2016), especially in clinical assessment situations where patients may feel ashamed of their limited health literacy (Parikh et al., 1996). In addition, it is possible that the questionnaire directly connects not taking medications with the perceived barriers to not taking them. This may have affected the comparatively higher number of barriers among participants with low medication adherence. Third, participants were recruited from a single medical center, and the participants may not represent all patients with T2D or in other healthcare settings. Also, people with limited health literacy may be less likely to be recruited, in that they may find it difficult to complete a self-reported survey (Althubaiti, 2016; Parikh et al., 1996). As such, findings should be considered exploratory in nature.

### Contribution to Mixed Methods Literature

The study provided a comprehensive understanding of barriers and factors of medication adherence through identification of specific psychosocial and interpersonal factors and exploration of corresponding contexts to these factors. Consequently, the results shed light on the usefulness of a theory-based mixed methods approach to empirical practice research. The explanatory sequential design appears to be a practical approach to addressing a holistic picture of patient factors associated with both medication adherence and health literacy.

Mixing quantitative and qualitative findings through integration highly corroborated one data set to another and provided more insights into a set of psychosocial factors relevant to medication adherence. An illustrative example is the differences in concerns about medication use, where we could not get more understanding of how patients handled this issue across different health literacy levels by analyzing quantitative data only. Coupled with excerpts from interviews, we gained a deeper understanding of how health literacy impacts participants’ problem-solving skills and concerns about medication use. For example, there was no statistically significant difference in patient-provider communication across clusters of different health literacy levels. However, the qualitative findings indicated different patterns of conversation between patients and their providers, such as conversation contents and counseling style. Hence, this finding paves a way for future research to explore the process of communication between patients and providers across different health literacy levels. Our study augmented a tight link between theory and methods, facilitated transparency and accountability of our results, and therefore increased the credibility of our overall findings.

In summary, using a mixed methods approach with theory-driven technique is feasible in healthcare research of complex phenomena (e.g., medication-taking). This study shows how beneficial it is to integrate mixed methods with theory-based approaches in healthcare research in order to enrich our knowledge of tailored intervention for improving adherence to medications.

## Conclusion

The findings underscore the complexity of medication adherence and the underlying patient factors. By using a mixed methods design, the results depicted various types of prevalent barriers that can be targeted in subgroups of interest when an individual-level barrier assessment is not feasible in practice. The findings further highlight the need to address patients’ psychosocial factors and patient-provider communication after accounting for their health literacy levels to improve patients’ medication adherence. For high-adherent patients, regular tracking of their medication-taking with an emphasis on their possible barriers to medication use would be sufficient. Special attention to improving self-efficacy among low-adherent patients with adequate health literacy should be in place. In addition, addressing both self-efficacy and concerns about medication use may be effective for low-adherent patients with inadequate health literacy.

## Data Availability

The raw data supporting the conclusions of this article will be made available by the authors, without undue reservation.
