# Supplementary material for: Investigation of Barriers and Facilitators to Medication Adherence in Patients With Type 2 Diabetes Across Different Health Literacy Levels: An Explanatory Sequential Mixed Methods Study
Source: Front Pharmacol. 2021 Oct 6;12:745749. doi: 10.3389/fphar.2021.745749 (PMC8527013; doi:10.3389/fphar.2021.745749)
Supplement: Supplementary file 1 [file DataSheet1.pdf]

Appendix S1: Questions for the interview

Opening question

Sometimes people stop or forget to take their diabetes medications. Has this happened to you?

General questions about patients experience

1. What are the most important things you do to take care of your diabetes?
   (1) What do you think the role of medications play on your diabetes?
2. What do you feel gets in the way of taking your diabetes medications?

Patient-provider communication

3. Many patients have trouble communicating with their doctor. How has your experience been with communicating with your doctor about your diabetes?
4. How does your communication with healthcare providers affect how you to take diabetes medications as prescribed?
   (1) What information does your doctors often talk to you?
   (2) What information do you want your doctors talk to you?

Questions for barriers to taking diabetes medications:

5. How confident are you in taking your diabetes medications properly?
   (1) Please explain what makes you feel confident you can take your medications.
   (2) How did your confidence affect taking your medications over the past 6 months?
6. What do you find the hardest about taking your diabetes medications as prescribed?
7. What problems or difficulties have you had that make it hard for you to take your diabetes medications as prescribed?
   (1) What do you think could be done about these problems or difficulties about taking your medications?
   (2) What possible solutions comes to mind?
8. What concerns do you have about taking diabetes medications as prescribed?
   (1) How did these affect your use of diabetes medicines over the past 6 months?
   (2) What would help you overcome your concerns?

Questions for facilitators of taking diabetes medications

9. Is there anything that makes the management of diabetes medications easier for you?
   (1) Please tell me more about what/how makes its easier.
10. What have you done in the past to help you take your diabetes medications as prescribed?
11. What could your providers do that would be most helpful for taking your diabetes medications as prescribed?
12. Is there anything that you'd like to share about your diabetes medications?

Appendix S2: Sociodemographic backgrounds of the participants in the quantitative phase (n = 205)

Variables	n (%)	Mean (S.D.)
Age		60.9 (10.20)
Sex		
Female	116 (56.6)	
Male	89 (43.4)	
Race		
Non-Hispanic White	154 (75.1)	
Others (Black, Hispanic, and Asian)	51 (24.9)	
Education		
Completed 8th grade or less	1 (0.5)	
Some high school	11 (5.4)	
High school graduate or GED	48 (23.4)	
Trade school	17 (8.3)	
Some college	50 (24.4)	
Associate’s degree or a 2-year college degree	27 (13.2)	
Bachelor’s degree or a 4-year college degree	35 (17.1)	
Master’s degree	12 (5.9)	
Doctor's degree	4 (2.0)	
Annual household income		
Less than 20,000	59 (28.8)	
Equal or more than 20,000	146 (71.2)	

Appendix S3: Comparison of psychosocial and communication factors between clusters

Variables (Mean ± SD)	Inadequate HL, high MA (Group A)	Inadequate HL, low MA (Group B)	Adequate HL, high MA (Group C)	Adequate HL, low MA (Group D)	Post hoc test results
Number of subjects	23 (11.2%)	33 (16.1%)	66 (32.2%)	83 (40.5%)	
Self–efficacy***	36.96 ± 3.02	30.70 ± 5.83	37.42 ± 2.96	33.47 ± 4.74	Group A > Group B and Group D<br>Group C > Group B and Group D
Concern beliefs**	13.26 ± 4.54	15.03 ± 3.75	12.45 ± 3.54	13.94 ± 3.39	Group B > Group C
Necessity beliefs	19.13 ± 4.90	19.85 ± 3.06	18.98 ± 3.42	18.82 ± 3.33	
Perceived barrier***	0	2.09 ± 2.85	0	1.88 ± 2.17	Group A < Group B and Group D<br>Group C < Group B and Group D
Provider-patient communication	9.73 ± 2.03	9.48 ± 3.17	9.56 ± 2.37	9.18 ± 2.64	

Note:

0 = no barrier was reported by this group of participants.

HL: health literacy; MA: medication adherence

$^{*}p < 0.05$, $^{**}p < 0.01$, $^{***}p < 0.001$
